# Operationalizing competency-based assessment: Contextualizing for cultural and gender divides

**DOI:** 10.12688/mep.19728.1

**Published:** 2023-10-11

**Authors:** Samar Ahmed, Fouzia Shersad, Arina Ziganshina, Mariam Shadan, Abdelmoneim Elmardi, Yousif El Tayeb

**Affiliations:** 1 Faculty of Medicine, Forensic Medicine and Toxicology department, Ain Shams University, Cairo, Cairo Governorate, 11488, Egypt; 2 Associate Dean Academic Affairs, Dubai Medical College for Girls, Dubai, United Arab Emirates; 3 Medical Education, Dubai Medical College for Girls, Dubai, United Arab Emirates; 4 Clinical Department, Dubai Medical College for Girls, Dubai, United Arab Emirates; 5 Biomedical Department, Dubai Medical College for Girls, Dubai, United Arab Emirates; 6 Clinical Department, Dubai Medical College for Girls, Dubai, United Arab Emirates

**Keywords:** equity, diversity, inclusion, EmiratesMEDs, competency-based medical education, programmatic assessment, contextualization, student progression

## Abstract

Following current trends, educational institutions often decide to use a competency framework as an overarching structure in their assessment system. Despite the presence of a common understanding of how different examinations can contribute to the decision on attaining a particular competency, a detailed mapping of the data points appears to be a challenging area that remains to be explored. Faced with the newly emerged task of introducing the assessment of the attainment of UAE medical students against the EmiratesMEDs competency framework, Dubai Medical College for Girls (DMCG) attempted to operationalise the designed concept in the assessment system considering the cultural and gender divide. We believe that health professionals who attempt to implement contextualized competency-based assessment could benefit from being acquainted with our experience.

The article offers a step-by-step guide on contextualized competency assessment operationalization, describing building the team, working with consultants and faculty development, estimating institutional assessment capacity, mapping and operationalizing the maps by using both human recourses and the software. We also offer the readers the list of enabling factors and introduce the scope of limitations in the process of developing the competency-based assessment system.

We believe that following the present guide can allow educators to operationalize competency-based assessment in any context with respect to local culture and traditions.

## Background

Assessment is undoubtedly one of the most significant components in health professionals’ education (HPE). On the one hand, passing examinations is a gateway to practice. In this sense, maintaining a rigorous assessment is a prerequisite for high-quality safe patient care ( Sutherland & Leatherman, 2006). On the other hand, testing provides trainees with the evidence about their actual performance required to inform future actions and individual development strategies. Therefore, the assessment must produce sufficient data to highlight the strengths and weaknesses of the candidate to generate suggestions for improvement ( Boud & Molloy, 2012). In addition, feedback from examinations should contain sufficient information on the candidate performance for potential employers ( Chandratilake *et al.*, 2009). 

According to contemporary vision, the optimal assessment system in HPE is coherent, integrated, and promotes life-long learning ( Eva *et al.*, 2016). In other words, data on candidate progress must be available throughout the training and practice continuum to ensure ongoing professional development and to allow justified decisions on one’s proficiency. Another important aspect to be mentioned is mutual accountability between the trainee, educational institution, and a healthcare system as a prerequisite for high-quality professional performance in the field and ongoing improvement ( Bordage *et al.*, 2013). The modern approach proposed to fulfil the listed criteria is known as programmatic assessment ( Van der Vleuten *et al.*, 2012). 

According to the programmatic approach, high-stake decisions on student progression in attainment of particular competencies must be informed by multiple data points ( Lindemann *et al.*, 2021). Following the current trends, educational institutions often decide on using a competency framework as an overarching structure in their assessment system ( Bok *et al.*, 2018; Upadhyaya *et al.*, 2021; Van Melle *et al.*, 2019). On practice it implies that information from individual assessment moments has to be aggregated across the domains or roles of the selected framework. Despite the presence of common understanding of how different examinations can contribute to the decision on the attainment of a particular competency, a detailed mapping of the data points appears to be a challenging area that remains to be explored. However, faced with the newly emerged task of introducing the assessment of the attainment of UAE medical students against the EmiratesMEDs competency framework, Dubai Medical College for Girls (DMCG) attempted to operationalise the designed concept in the assessment system.

UAE has emphasized the importance of empowerment and knowledge acquisition for women and moved beyond the older tradition where women were primarily homemakers. The contribution of Dubai Medical College for Girls (DMCG) in this direction is noteworthy as it is the nation’s first private medical school and the only one solely for women since 1986. Though formal education of girls started way back in 1960, the social structure still mandated a gender-segregated education whether it was in schools or universities for several decades after. School and university policies issued by the Ministry still utilize decrees articulated in the Arabic language and in line with Islamic law, which imposes certain barriers, especially for authentication of online examinations. Another unique characteristic of the country is its diversity owing to the large proportion of the expatriate population. On one end of the spectrum is the social structure which guards the females and on the other end is the diversity brought in by the predominantly expatriate population. This diversity means that every cohort contains approximately 20 nationalities. 

The EmiratesMEDs, the National Competency Framework for Medical Education was released by the Commission of Academic Accreditation, aimed at tailoring the existing learning outcomes of medical programs to the needs of the UAE society and healthcare system. EmiratesMEDs was created through an iterative process of reviewing the internationally accepted frameworks for Competency based Medical Education and utilizing the feedback of all stakeholders. The EmiratesMEDs framework consists of nine thematic roles described as Core Competencies and each subdivided into enabling competencies. ( Commission for Academic Accreditation, 2023) 

These thematic roles and their enabling competencies are specifically framed to suit the future needs and the long-term vision of the nation. The unique demographics, healthcare needs, and explosive growth of information in the Nation and region were also given special consideration. The major characteristics which make this framework stand apart are the contextualization with the diverse and expatriate population of UAE, the UAE legislation, the specific health needs, the culture of UAE and the underprivileged section of the population of UAE. ( Commission for Academic Accreditation, 2023)

Unlike most traditional medical schools around the world, DMCG can be considered a truly international college. Attracted by the opportunity to learn medicine in a safe and welcoming environment sensitive and inclusive to the religious and cultural specifics of the East, families not only from Gulf states but also from other corners of the world choose DMCG for their daughters. Along with the beauty of having a variety of cultures in a single school, such diversity in student populations creates certain challenges in selecting the right approach to each and every trainee regardless of their previous educational and geographical background. We also acknowledge that some of our future female physicians might need additional support when away from families in a foreign country, studying in English at their junior age. This paper elucidates how key strategies for assessment of a competency-based system have been implemented through policies underpinning gender-specific regulations aligned with Islamic values.

We believe that health professions’ educators attempting to implement competency-based assessment might benefit from being acquainted with our experience.

## Aim of the work

This work aims at offering a guide for schools looking to operationalize a contextualized competency assessment framework within their assessment system.

## Stages of development of the assessment plan

### Predevelopment

***1. Setting up the team***

A group of assessment leaders in DMCG, with diverse expertise in medical education and in assessment, teamed up to work towards the restructuring of the assessment system in DMCG to make it suitable for the new transformative changes taking place in the institution (the new curriculum) and UAE (the new doctors competency framework - the EmiratesMEDs). The team included the associate dean of academic affairs, the head of the medical education unit, the head of the assessment committee, and the assessment coordinator. It should be noted that the members have a diverse professional background, having previously practiced in educational institutions of Northern and North-East Africa, Eastern Europe, and South Asia. However, all assessment developers were at least previously exposed and familiar with Islamic values and culture. We believe that one of the assets is diversity in gender, age, and seniority among the team members. While most of the team members were women of prior leadership and executive positions in other places in the world and that have experienced a capped ceiling for their executive scope yet the collective brains and capacities were found to be of tremendous impact since, the organizational culture fosters inclusivity in participation in decision making, shared process ownership, and collaborative leadership for all relevant professionals. The team identified what needs to be done through a number of meetings and casual discussions and then agreed on how it could be done.

***2. Consultations and faculty development***

To ensure efficiency in both the process of restructuring of the assessment system in DMCG and its future implementation, the team communicated with institutions that have expertise in competency-based medical education and programmatic assessment. The team held consultation meetings for the purpose of benchmarking, consultation, training, and use of software in competency assessment. To ensure satisfactory engagement of stakeholders, particularly faculty, during the planning and designing phase, and their future buying-in during the implementation phase, a number of faculty development activities were organized. This included a workshop on programmatic assessment and a number of training sessions on the effective use of examination software provided by the vendors of the software.

When settling for decisions derived from consultations and external training, it was important to be selective about the strategies that worked or were bound to work in a culture that was mainly female driven and a vision that honoured the Islamic culture. This was materialized in decisions to prioritize issues that were pertinent to ensure vigorous follow up of students that started at a maximum and ensuring that the amount of freedom and student-centered education increased with the progress of years of study. Concepts like the use of student portfolio were brought forward as an early educational necessity and thus a more important set of practical tips that were prioritized in any collaboration, consultancy and training. 

***3. Study of the current institutional assessment capacity***

As part of the planning process for newly introduced operations, the institutional capacity assessment is done. The assessment's goal is to ascertain whether the Executing Agency has the resources required to execute the project. The team implemented an institutional capacity assessment to determine the readiness of the school for the contemplated restructuring of the assessment system. It aimed at identifying gaps and deficiencies in the existing technology and the capacity of the stakeholders and the governance system. The McKinsey 7S framework was used for that purpose ( McKinsey, 2020). The Structure, Strategy, Systems, staff, skills, style, and shared values were assessed. The malignments, gaps, and deficiencies were identified and addressed. The existing assessment policy was reviewed and is still undergoing iterations. It was decided that the use of the learning management system and other technologies used at DMCG, e.g. Competency AI should be optimized. It was also decided that some additional software applications are needed, namely a student information system and an efficient assessment suite, ExamSoft™, are to be acquired and integrated into the assessment system. A faculty development program is planned and is ready for implementation.

There were two important factors identified as the core of the context in which the program was to be conducted which were the fact that this college was an Islamic Women’s college. This meant that the nature of the student body was unique and had a set of separate capacities that needed to be tailored into any assessment plan decisions. These issues were transformed into a set of implications that were later on addressed in all decisions. These were:

- Students came straight from high school and being females in a universal Islamic culture entailed that the degree of exposure that they received until coming to college was very limited.
- Students were used to a more authoritative educational culture and the amount of responsibility they had previously taken for their own education is very limited.
- Students came mostly from homes that prioritized their education and where a girl’s education became more like a family project where parents and siblings became highly involved in educational decisions.
- Despite the international nature of the student body, being an Islamic school with a vision that held religion at the forefront homogenized to a great extent the nature of the students and this was important to address in every decision to ensure that students became well versed with the Dubai culture of openness and inclusiveness.

### Mapping

***1. Mapping the course learning outcomes with Program learning outcomes and enabling Competencies and EPAs***

This was done through a number of meetings where this map was revised and reiterated multiple times. This map became the master sheet where all course learning outcomes were standardized. It was then used as the sole reference for all enabling competency and EPA mapping to the curriculum. The master sheet sample can be seen in Table 1.

**Table 1.** Mapping the course learning outcomes with Program learning outcomes and enabling Competencies and EPAs for DMCG MD program.

Course Learning Outcome (CLO) code	CLO	Program Learning Outcome	EmiratesMEDs Competency areas									
			ME	EBS	PC	C	CIL	P	HS	SPE	SA	EPA

EPA mapping was found to be highly populated in course learning outcomes of phase 3 (clerkship phase). Nevertheless, some EPAs were mapped sporadically with course learning outcomes in earlier stages. This is since the curriculum is initially a clinical presentation curriculum.

Mapping of enabling competencies to Course learning outcomes was done in a series of meetings with subject matter experts. The first meeting was planned to orient them with the meaning of each of the enabling competencies. The second meeting was directed at aligning each of the CLOs with an enabling competency and the third meeting was planned to allow for peer feedback and recommendations on the initial map.

The final map was revised in a fourth meeting and the outcome was approved by the necessary councils with student involvement ensured.

***2. Mapping of the competencies to the courses addressed***

Another exercise was done where the Competency area was mapped against the courses that will address it. This map was important to start setting up the competency assessment plan. The map can be seen in Table 2.

**Table 2.** Mapping EmiratesMEDs Competency areas to the courses for DMCG MD program.

Course Name	Course Code	EmiratesMEDs Competency areas									
		ME	EBS	PC	C	CIL	P	HS	SPE	SA	EPA

Mapping competencies to courses was an important step that ensured that some of the courses in the study plan were addressed properly with the enabling competencies despite the fact that the credit load of these courses might not have been major. This appeared in courses like Islamic studies, Islamic Fiqh, UAE society, Communication skills, Medical ethics in phase one and courses like Women and Health in phase three. This was important to ensure that emphasis was placed on the courses that addressed directly the culture of the context.

***3. Teaching Learning Assessment Matrix***

While designing the courses and finalizing the course a form was designed where each course coordinator mapped the Assessment methods used in their courses Example can be seen in Table 3. This was verified against the announced assessment strategy. This was a step necessary to align the methods and to unify the used language.

**Table 3.** Mapping courses with assessment methods for DMCG MD program.

	Assessment															
Course Code	Multiple choice questions	extended matching questions.	Spotter examinations	OSPE	OSCE	DOCE	Patient logs	case-based discussion	directly observed practical skills	mini clinical evaluation exercise	evaluation of clinical events	multi-source feedback	Student project and assignments	In-Class Assessment	Quiz	Student Participation
**21CS101**	1	1											1	1		1
**IHB102**	1	1	1										1	1	1	1
**EMS103**	1	1											1	1	1	1

This was later imported into an assessment planning document where the percentage of each assessment method was entered and the week in which the assessment was to take place was also mapped in this stage.

***4. Program Learning outcome course mapping***

A Program learning outcome to course map was developed where the Program learning outcomes were listed and identified as to where they were addressed in the courses as it is shown in Table 5. The information to support this was extracted from the CLO PLO Enabling competency Master sheet and double checked against the information in the course syllabi.

***5. Course Assessment map***

Meetings were done to identify the percentage of continuous assessment versus the final assessment. The concept was to place more emphasis on the summative form of assessment. It was agreed to plan assessment for phase 1 and 2 different than phase three. More percentage was given to final assessments in phase 3 where forty percent of assessment only was done in a continuous fashion throughout the rotations. This was the opposite to what was agreed on for phase 1 and 2. The reason for this was the fact that much emphasis needed to be placed on the final exit clinical exam that was a part of the program assessment plan and that was required for student applications for internship opportunities. The example of the distribution of marks can be seen in a Table 6.

A course assessment plan was added to each course syllabus. This included the method of assessment, time of assessment and the percentage which it constituted towards the course final grades. This was done using a shared excel file that allowed only selection from a drop-down menu of assessment methods that were prior approved in the TLC matrix. This was done with the aim of limitation of variation of language when the assessment plan was set. An overview of the shared file can be seen in Table 4.

**Table 4.** DMCG MD program course assessment plan.

Course Code	Assessment Method	percentage (Weight)	Week
21CS101	In-Class Assessment	10	Continuous
	Student participation	10	Continuous
	Student assignments	10	Continuous
	Written examination (MCQ, EMQ)	30	Week 8
	Written examination (MCQ, EMQ)	40	Week 16
IHB102	In-Class Assessment	5	continuous
	Student participation	5	continuous
	Quizes	20	continuous
	Student assignments	10	continuous
	Written examination (MCQ, EMQ)	20	9
	Practical Examination (Spotter)	10	16
	Written examination (MCQ, EMQ)	30	16

**Table 5.** 

Course Code	1. Demonstrate knowledge and comprehension with substantive depth in areas of core biomedical, psychosocial, and clinical sciences.	2. Apply biomedical, psychosocial, and clinical sciences knowledge in the clinical context for promotion of health, prevention of disease, and the management of common clinical conditions within the framework of ethical and legal regulations as an undifferentiated general medical practitioner and in preparation for future specialist training.	3. Utilize interpersonal, communication and clinical reasoning skills to interview and elicit a patient's medical history and to communicate effectively with patients in contexts other than information gathering, caregivers, and the other members of healthcare team, within the context of cultural awareness.	4. Apply clinical and technical skills to perform physical examination and basic clinical procedures	5. Deliver patient care that is patient-centered, compassionate, appropriate, and effective for health promotion and health problems management.	6. Engage in research and other scholarly activities, and critically analyze existing literature to apply it for the practice of evidence-based medicine	7. Demonstrate continuous self-improvement, innovation, entrepreneurship, and lifelong learning abilities	8. Demonstrate an awareness of the system-based practice approach to patient care considering healthcare contexts locally and globally	9. Demonstrate the ability to meet the health needs of patients and UAE society, through the promotion of community engagement and social accountability values
21CS101	I						I		I
IHB102	I	I							
EMS103	I	I					I		I
UES104	I		I						I
WAH105	I	I				I			
PPS106	I	I				I	I		
ILS107	I,M	I	I						
THE108			I, R	I	I		I		
PHP305	I,R	R							R
MIS306	I,R	R							
CPS307	I,R	R	R	I		R			
RKT308	I,R	I			R	I, R			
GIS401	I,R	R	R					R	
HAN402	I,R	R							
Percentage of courses addressing the PLO	80.3	72.5	51	31.4	35	21.6	19.6	15.7	23.5

**Table 6.** Distribution of marks for a course for DMCG MD program.

#	Assessment methods	Marks	Due Date
**1**	In-Class Assessment	10%	Continuous
**2**	Student participation	10%	Continuous
**3**	Student assignments	10%	Continuous
**4**	Written examination (MCQ, EMQ)	30%	Week 8
**5**	Written examination (MCQ, EMQ)	40%	Week 16
**Total**		100%	

The amount of detail that was planned in this step was tailored to the assessment driven environment the students were coming from and the degree of parental involvement in follow up. Fleshing out the assessments that were planned for every year and the amount they contributed to the final mark remained an important point that needed to be fulfilled in this transitional stage where decisions and parental follow up remained tied to marks.

### Operationalizing the maps

***1. Revision of assessment policy and progression plans***

One of the important aspects to consider when introducing changes to the existing assessment system is ensuring alignment with the relevant policies and procedures within the organization. The decision on initiation of competency attainment evaluation is likely to result in introduction of new examination types, tools, and technologies. Such innovations require modification of the governing documents. The policy must be updated with the most recent templates of course and exam blueprints, rubrics, and checklists, designed specifically to address the attainment of all competencies. It is equally important to specify particular assessments and their weight in evaluating competency attainment. All terminology used must be unified and clearly defined.

Another area to be documented is the role of competency attainment in making decisions about student progression. In this regard, the policies that govern student progression were revised to ensure that there was enough flexibility to allow the use of competency attainment in decision-making. A decision was made to incorporate data from competency attainment in the student portfolio as a transitional phase and to keep all progression decisions based on student grade attainment and passing criteria as per the accepted standard setting. This was planned on a grade point average system according to the accepted grade point average cutoff of two for all courses. 

***2. Establishing functional committees***

To prepare the institution for the coming renovated plan of assessment, it was important to ensure the assessment back bone of the institution was fit for purpose. Given the difference in the potential utility of the examination data points in making progression decisions, it was necessary to ensure that there was an independent body in the college capable of providing a birds eye view on the competency progression of students and capable of translating the different data point entries into a decision. This was the rationale behind the formation of the competency assessment committee (the progression committee). This committee was formed of the heads of the academic departments, the chair of assessment and assessment experts. The mandate of this committee included designing the assessment plan for the adopted competencies, studying data collected within the competency assessment plan and studying student portfolio information. This committee was chaired by the associate dean academic affairs and gets its approvals from the college council.

Course committees were appointed, and a course coordinator was tasked as the main contact point for each course. Phase coordinators were also appointed. The roles and responsibilities of each of these layers were referred to in the revised policy.

A coordinator for student portfolio was appointed with the plan to report to the competency progression committee.

***3. Setting assessment principles***

*Methodology of development of the assessment principles*

A task force of assessment experts was brought together in a set of three meetings where the issues related to the competency assessment principles were discussed. A list of principles was generated as a draft and it was circulated for refinement twice among them until consensus was gained and the principles were then approved by the council and published.

*Assessment principles*

a) Selection of data points for Competency evaluation is done by collective inputs from medical education experts based on decisions on importance.

b) Competencies are addressed in multiple courses in the form of teaching or training whereas, selection of valid datapoints for assessment of competencies does not necessarily include all those courses.

c) The weight of student knowledge acquisition is higher in Phase 2.

d) The weight by which the patient care competency is addressed is higher in Phase 3.

e) Data points for assessment of professionalism are selected based on weight by which it is addressed in different courses.

f) Data points are generally calculated based on course credit hour value with some modifications considered for different phases, as follows:

i. Assessing medical expert competency, credit hours of phase 2 courses were calculated by multiplying by a factor of 1.25.
ii. Assessing medical expert competency of credit hours of phase 3 were calculated by multiplying by a factor of 0.75.

g) Examination blueprints for selected exams will be designed based on competencies they address.

h) In terms of the assessment of certain thematic roles, it was decided that not all courses that contribute to the attainment of a particular competency will be considered. It was agreed that the data points would not be included in the competency evaluation plan if the credit that addresses thematic role in a particular course is less than 20%. Such an approach was applied to avoid redundancy and to ensure adequate contribution of all relevant courses to decisions of the student progress in each thematic role.

i) The assessment plan was designed in such a way to assure alignment of program content, teaching, assessment and learning outcomes.

***4. Calculating quantifiable data points for the program***

*Methodology of development*

The subject matter experts were invited to a series of meetings where they gave their view on the examination data points where there could be an assessment of each of the competency areas. This was done guided by the initial CLO enabling competency map ( Table 1). The competencies addressed in each course were laid out then the map was reversed where each competency area identified was tabulated together with the courses where the competency area is addressed.

This helped focus the competency assessment to a number of courses rather than having to assign data inputs from the whole program towards assessment of each competency area ( Table 7)

**Table 7.** EmiratesMEDs Thematic roles development structure example. Name of thematic role: Self and Profession Enhancer.

Course	21CS101	PAE209	HLE507	OGW601
Share in competency development	% towards competency attainment			

*Quantitative competency assessment tables*

This was designed for every competency identifying the actual percentage of each assessment data point contribution to the total competency assessment. An example can be seen in Table 8.

**Table 8.** Example of Quantitative EmiratesMEDs competency assessment table at DMCG.

Course	RKT308		BIS404		RKT409		EBM603	
Share in competency development	30%		10%		30%		30%	
Assessment method	source	weight	source	weight	source	weight	source	weight
	written	10%	written	10%	assignments	10%	assignments	10%
	project	20%			Written week 8	10%	Written week 8	10%
					Written week 16	10%	Written week 16	10%

Designing the quantitative tables was important for the quantitative reports that were to be developed. The nature of the students and parents that were addressed dictated that a considerable degree of quantification was required to understand the student progress. This emerged from the years of closely supervised education that a Moslem girl passed through all the way up to college. Untying the knot and emerging into qualitative and global understanding of progression needed to be approached with extreme care. Thus quantification of competency progress was planned from year one to allow for a longitudinal follow up strategy that will be more understandable and palatable for students and their parents.

***5. Coding data points***

In order to develop the student grade book and the competency assessment report for each student, each data point was coded with a strict coding system where the course code was added first then followed by type of assessment serial number if necessary and finally the week it is delivered in.

***6. Designing a master assessment plan document***

This document was designed by the assessment team where for every coded data point there was a plan including the time when it would be collected, method of collection, percentage it comprised within the total course mark, time when it would be evaluated and when feedback would be given. Some items were marked if they were to become a part of the student portfolio. The master assessment plan sample can be seen in Table 9.

**Table 9.** Example of master assessment plan for DMCG MD program.

									THEMATIC ROLES OF EMIRATESMEDS									Portfolio
Course Code	Assessment Method		Data point Code	Number of exam items	percentage (Weight)	Week	**CLO**	Requirement of Blueprint/rubric	ME	EBS	PC	P	C	CIL	HS	SPE	SA	

***7. Designing Course assessment blueprints***

For every course the assessment blueprint was designed to reflect the content of the master assessment plan and flesh out the details of each course. This document was designed to be handled by the course coordinator during implementation. A sample of the course assessment blue print can be seen in Table 10.

**Table 10.** Example of course assessment blueprint for DMCG MD program.

Assessment Task Code	CLOs	Date	No. Of items	weight
101ICA1,	21CS101/01, 21CS101/02	3		2
101ICA2,	21CS101/01, 21CS101/03, 21CS101/04	6		2
101ICA3,	21CS101/01, 21CS101/03, 21CS101/04	9		2
101ICA4,	21CS101/01, 21CS101/03, 21CS101/06	12		2
101ICA5	21CS101/01, 21CS101/03, 21CS101/09	15		2
101SPA1,	21CS101/03, 21CS101/04, 21CS101/06, 21CS101/07	10		5
101SPA2,	21CS101/03, 21CS101/04, 21CS101/05, 21CS101/08	16		5
101SAS1,	21CS101/03, 21CS101/04	4		2
101SAS2,	21CS101/03, 21CS101/04	7		2
101SAS3,	21CS101/03, 21CS101/04	10		2
101SAS4,	21CS101/03, 21CS101/04, 21CS101/07	13		2
101SAS5	21CS101/01, 21CS101/02, 21CS101/03, 21CS101/04, 21CS101/05, 21CS101/06, 21CS101/07, 21CS101/08, 21CS101/09	16		2
101MCQW8	21CS101/01, 21CS101/02, 21CS101/03, 21CS101/04	Week 8	20	30
101MCQW16	21CS101/01, 21CS101/02, 21CS101/03, 21CS101/04, 21CS101/05, 21CS101/06, 21CS101/07, 21CS101/08, 21CS101/09	Week 16	30	40

***8. Design of student portfolio***

The electronic portfolio (e-portfolio) is a well-established method for effectively evaluating student achievement within the context of competency-based medical education (CBME) ( Chertoff, 2015). This student-centred activity empowers students by giving them control over their learning trajectory, instils a sense of ownership, and serves as a foundation for lifelong learning ( Shrivastava & Shrivastava, 2021). As these skills equip the students to transition smoothly from school to college life, the portfolio was integrated into the foundation year curriculum.

The development of the student's portfolio was based on in-depth analysis of guidelines and the experiences of leading institutions worldwide ( Shrivastava & Shrivastava, 2021; Van & Driessen, 2009). The e-portfolio assesses undergraduate medical students in nine competency areas defined by Emirates-meds. Through the identification of relevant data points in the undergraduate curriculum, the e-portfolio captures and visually presents competency attainment data over time.

The e-portfolio was preferred over paper-based systems due to its robustness, regular student engagement, user-friendliness, ability to generate large amounts of high-quality data, and superior data manipulation capabilities. These web-based portfolios have been proven to be inclusive, equitable and accessible for both students and mentors, promoting their wider acceptance and engagement ( Driessen *et al.*, 2007). Furthermore, it provides accurate assessments of students' progress in achieving competencies over time ( Chertoff, 2015).

To ensure that the e-portfolio facilitates learning, the design incorporated periodic assessments, academic supervision, and feedback, recognizing and acknowledging students' efforts in maintaining the portfolio ( Shrivastava & Shrivastava, 2021). This approach aims to motivate students to continue using the e-portfolio and encourages reflective thinking on how to improve it. Also, regular staff development workshops were conducted to support advisors in understanding and meeting the diverse learning needs of students ( Kelly & Moogan, 2012).

***9. Design of the annual grade book***

All data point codes were tabulated with their relative percentage towards course grade was mentioned. Course totals were added and the interpretation to grade point average was also added. ( Table 11)

**Table 11.** Sample of the grade book for one course of DMCG MD program.

Course Code	21CS101															
Data point Code	101ICA1	101ICA2	101ICA3	101ICA4	101ICA5	101SPA1	101SPA2	101SAS1	101SAS2	101SAS3,	101SAS4,	101SAS5	101MCQW8	101MCQW16	Total	Grade Point
Percentage from course total	2	2	2	2	2	5	5	2	2	2	2	2	30	40	100	0–4

https://examsoft.com/

***10. Design of the Test Tagging tables (TTT)***

For every coded test item, a test tagging table was designed by the course coordinator in collaboration with the assessment team. This table mapped every test item to the course learning outcome and the enabling competency. This was done and cross referenced with the master assessment plan. This step was an additional step to ensure that setting up the item bank for future examinations was done as per the required tagging for enabling competencies. This would ensure proper reporting for the competency attainment by the end of the academic year in a collective manner and for the reports that were required for academic advisory processes throughout the year.

### Mobilizing and adapting technology

Digital examinations have become indispensable tools to automate and streamline execution of examination plans. At a minimum, technological tools make it easy for students and helps faculty to better utilize exams and results for student improvement while saving time and manpower. Technology tools were adapted for the Islamic context in a girls-only college.

Currently, technology continues to achieve unimaginable horizons to create innovative practices in assessment and connect learners and institutions ( Alexander *et al.*, 2019)

***1. Selection of examination software***

A recent article on technology enhanced assessment states that the key foci for an examination software are enhancing authenticity, engaging learners, improving design and optimizing recording and tracking of learner progress ( Fuller *et al.*, 2022). The AMEE Guide No 154 guides institutions on ways to overcome challenges in choosing and developing mobile technology to conduct workplace-based assessment and assess EPAs ( Marty *et al.*, 2023).

The over-arching aim is to promote continuous assessment by using technology tools which capture and package large amounts of data into a comprehensible format. The DMCG assessment plan called for technological solutions that could package large amounts of assessment data into concise and authentic information which can be used for evidence-based progression decisions with minimal disruption of learning.

The DMCG Strategy for assessment is to keep online modes of examinations to a minimum, keeping in consideration, the limitations posed by religion, families and cultural norms particularly for female students. In 2020, during the pandemic, the Ministry of Education provided instructions that all security features such as continuous video-recording and monitoring of head and eye movements should be implemented by all universities for high-stakes examinations. As soon as the pandemic abated, DMCG re-instated the regulation that all exams will be conducted face-to-face. This was necessitated due to the considerations for Islamic beliefs on exposing the face and ears.

The role of the parents in funding and supporting the students indicates that they have a right to make decisions for the students. The family dependency is another factor ingrained in the culture of UAE and therefore, information on student progress is made available to the parents. In studies reported from Saudi, parents were the most powerful influencers in female students’ decisions and therefore, involvement of parents have been factored by DMCG ( Rozek *et al.*, 2017).

The following criteria were considered in the selection of the examination software:

- Versatility of the Examination Platform
- Safety and Security of the Exam Platform
- Accessibility and its control
- Scalability for Better Reach
- Easily Integrable
- Compliance and Privacy Protection

Other issues which were considered included finances, infrastructure, privacy, disaster recovery features, data access and data ownership.

From this experience, we have summarized the major requirements as follows:

a. System capabilities:

Several inherent system functionalities are identified as essential qualities for a reliable examination software.

- Flexibility – The system should be flexible enough to evolve as priorities change and the architecture should be scalable and adaptable to unique student requirements.
- Scalability – On-demand scalability and future-proof architecture will allow flexibility for continuous improvement.
- Disaster Recovery – Disaster recovery capabilities should be built-in and sturdy.

b. User interface

A major enhancing feature is user-friendliness. Ease of use, single sign-on capabilities, accessibility and requirement for training are other important parameters which ensure compliance and buy-in. Successive cycles of assessments and remediations are made possible by an interface which allows easy tracking of progress and detection of areas for improvement.

c. Integration capabilities

The system should be integration-friendly so that it can integrate with the other solutions used for education within the campus and can support the objectives of a connected campus. 

d. Data management and security

Comprehensive and robust data security and privacy protection is a mandatory requirement for assessment software. A powerful data management tool with accurate analysis of data should help to make decisions which improve student competencies.

e. Access Control and compliance with standards

The system should control access to information based on roles, offer field-encryption and data-masking, and comply with industry regulations and institutional standards.

f. Vendor relationship

Recommendations for selection include looking at the capabilities of the software and vendor relations. It is important to maintain positive relationships and communication with vendors who understand the institution’s culture.

g. Track-record

Sharing best practices and success stories with other experienced users will help ensure a good track record and troubleshoot proactively. Participation in communities of practice and learning from experienced user-institutions has shown to be of great value.

h. Mobile-based software to capture workplace-based assessments

Technological tools help in packaging the multiple assessment data segmented in different dimensions such as competencies, course learning outcomes, systems and threads. ( Marty *et al.*, 2023) The large amount of data obtained from multiple datapoints is packaged by the examination software in such a way that the Competency Committee can make accurate decisions on readiness to take the summative examinations.

***2. Adapting software***

The major software solutions identified for use at DMCG for student assessment are ExamSoft EXAMSCORE and Competency.AI (DOT) software systems. All assessments are performed on these software solutions and the gradebook is displayed in the Learning management system (LMS) and the Student Information System (SIS).

The interplay of these software solutions and the use of these tools for implementing the assessment plan of the MD program are discussed below:

*Examination Software System (ExamSoft™)*

The Examination Software System (ExamSoft™) is used for all written examinations where the student performs the examinations directly on the software. Multiple choice questions (MCQ) and Quizzes are examples of assessments directly on this system and automatically graded.

The functions performed on ExamSoft™ include Question bank preparation, validation of the exam questions, categorization, preparation of an examination question paper and moderation. The software provides a mechanism for capturing the student performance with a high degree of data security and performs automatic grading based on the pre-determined rubric and grading system. This step is followed by post-examination moderation, item analysis and categorization which help in analysis at different levels. Open-ended assessments with essay type questions and assignments can also be scored on this software. Instructors can choose whether or not to display results to the students.

The data to be entered into the ExamSoft™ are

1) Courses: The courses are listed under each program.

2) Question Bank: The question banks are created as a separate chapter and the validated questions are stored under each course with the statistical data of item analysis.

3) Categories used at DMCG:

i) CLO (Course Learning outcomes)
ii) PLO (Program Learning outcomes)
iii) Competencies & Enabling competencies
iv) Difficulty level

4) Reports: The reports are categorized as assigned and are displayed for faculty and staff who have access. It is also displayed to students on a special student portal. The final results with statistics will be pushed to LMS and SIS from ExamSoft™.

*Plug-in for Student–Centered Objective Rubrics Evaluation (ExamScore™)*

Student participation and in-class assessment scores can be entered through direct entry of scores by instructors twice a semester using a plug-in of ExamSoft™ called Student–Centered Objective Rubrics Evaluation (ExamScore™). Data can be entered into ExamScore™ by faculty/assessors either using the iPad or desktop PC.

The clinical examinations such as OSCE, OSPE, Student assignments, Projects and Practical spotter results are graded by assessors in real-time or from recorded patient encounters using iPads on ExamScore™ rubrics/checklists.

*Direct Observation Tool or DOT application (Competency.AI™ software)*

The direct observation tool (DOT) application is a mobile-based tool from Competency.AI™ to perform all workplace-based assessments (WPBA). WPBA results are used to assess achievement of EPAs and competencies to track progress and need for remediation.

Direct observations of students documented as workplace-based assessments are done by the clinical preceptors at the bedside and will be entered directly to the Competency.AI™ software through mobile application. The application is activated on the preceptors’ mobiles, and they are trained in the use of the software with unique usernames and passwords. The preceptors may also use the QR codes generated by students. Use of QR code will help preceptors to do their assessment in real-time and avoids the need for returning to it at a later stage. The assessment will be initiated by the student who generates the QR code for the preceptor, so that the checklist pops up in the preceptors' mobile phones.

The advantages of Competency.AI™ is that it is a very user-friendly direct observation tool for preceptors to use. It has capabilities of meticulous mapping of checklists and rubrics with competencies of EmiratesMEDs. The direct observation scores can be segmented at different levels such as competency, assessment type, assessment, system and thread. The checklists will help in evaluation of students' attainments of EPAs. These are in turn mapped with relevant competencies. The reports show the performance of each student against the competencies, EPAs, systems or threads in interactive numerical and graphical data. This is accessible to the mentors and advisors and the competency committee for making decisions on the progression of the students. The students requiring remediation can be identified for relevant action. At a program level, cohort performance can be segmented based on the same parameters.

*Visualization of the data*

Results are available for students to track their progress on personalized dashboards of LMS gradebook and SIS. Students can visualize detailed scores on the student portal of ExamSoft™.

On ExamSoft™, detailed numerical and graphical display of data segmented by categories are available on the personalized dashboards of faculty members/instructors. Administrators have the provision to control the access of instructors and each instructor can in turn control access to students to view their data on ExamScore™ and ExamSoft™ platforms. The final results and accompanying statistical data will be pushed into the LMS and SIS by the ExamSoft™ software. This will be displayed in the grade books of LMS and SIS.

The Competency.AI™ software provides actual data and graphical visualization of results in highly interactive dashboards for tracking learner progress. WPBA will be assessed on Competency.AI™ for fifth year and sixth year students and will be done and displayed to individual students and advisors. At DMCG, currently, WPBA results are displayed in the Competency.AI dashboard and are not integrated with SIS or LMS. Competency committee uses data for progression and as requirements for graduation.

***3. Faculty and staff development for the use of software***

The organizational change and digitization require effective faculty training to ensure that the assessment process is resilient and to create a sense of belonging and acceptance of the change.

Faculty development on the use of each of the tools is imperative to be initiated as early as possible and should be done in a step-by-step manner to keep them motivated. This will be a part of the change management process and we need to ensure that they learn the right skills for the right reasons. The indirect outcomes of faculty training are to motivate them and avoid burnout, nurture leadership, foster mentoring and promote student experiences. Training should address the gender issues related to Islamic traditions. IT professional training on Portal administration precedes faculty training for both software solutions.

*Faculty and staff development for ExamSoft™:*

The software vendor of ExamSoft™ provides an onboarding program. This contains sequential online training modules on Portal administration, Question creation, importing, Assessment building and exam day preparation followed by Reporting Review and post examination discussion. This happens over a period of two months.

*Faculty and staff development for Competency.AI™:*

Competency.AI™ training has been provided to the administrative staff and coordinators. The advantage of the software is that it requires minimal training of preceptors. However, very short orientation sessions and review sessions have been provided by regular meetings. An instruction manual has been created for this purpose.

Faculty training will continue and will progressively include more members as core assessment team members learn and become champions for change. In addition to the training videos, the vendors have provided a help-link within the software, so that users can troubleshoot as needed.

***4. Integration of individual software***

**Figure 1.**
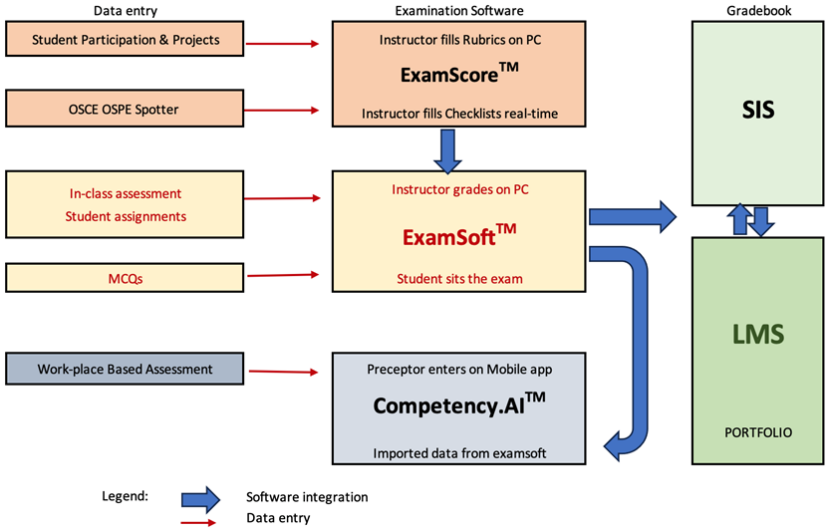
Flow of data from entry point, through exam software to Gradebooks.

The ExamSoft™, ExamScore™ and Competency.AI™ software systems are planned to enable specific assessment types. The assessment data is fed into the LMS and SIS where the gradebook templates are located. These are shown in Figure 1. Flow of Assessment Data and Integration of Software.

ExamScore™ contains rubrics which are used by instructors to evaluate students on their performance and participation, and these are entered using rubrics directly using their desktop/handheld devices. The OSCE and OSCPE are graded on ExamScore™ during the exam on the iPAD.

The ExamSoft™ software captures all written examinations which are mostly in the form of MCQs directly while the student sits the examinations, and they are automatically graded in real-time. These exams are planned to be conducted face-to-face to ensure reliability and validity. In addition to the MCQ type, essay type questions and assignments are scored on ExamSoft™ by instructors on their PCs as per rubrics.

Direct observations of students or workplace-based assessments are entered by the clinical preceptors at the bedside and will be entered directly to the Competency.AI software through mobile application.

The results are displayed on student portals of ExamSoft™, Competency.AI™, LMS and SIS. Further integration of ExamSoft™ with Competency.AI™ is planned to be done when data from WBPA is implemented and at that stage, all the data from ExamSoft™ can be displayed on Competency.AI™.

### Setting evaluation plans

In a competency-based education setting, student assessment is viewed as part of the educational design. The assessment culture has shifted its focus from psychometric measurement of learning to an assessment culture which is aimed at stimulating self-regulated learning. Therefore, evaluation of quality of assessment should focus on whether the assessment methods are fit for the purpose they are intended.

One of the frameworks to assess Quality of Assessment has been modelled around the Quality Pyramid of Assessment which is comprised of Assessment Policy, Assessment program, Assessments, Assessment tasks and Assessment literacy which are supported by a robust Assessment organization. ( Sluijsmans & Struyven, 2014) Another example of a framework to evaluate the QA of Assessment is the wheel of competency assessment ( Baartman *et al.*, 2006)

The UAE national accreditation systems requires the use of a variety of appropriate assessment tools aligned with course learning outcomes and maintaining appropriate rigor through moderation at multiple levels. Accreditation also requires clear strategy, policies, standards and guidelines for academic and administrative processes which are approved, disseminated and implemented. 

*QA criteria adopted*

The following Quality Assurance (QA) criteria were adopted

1. The cognitive complexity and rigor of assessment tasks should be appropriate.
2. Assessments should be meaningful and beneficial for students and teachers.
3. Assessment methods must be fit for the purpose of enhancing learning.
4. Assessment should be fair and inclusive.
5. Assessment criteria should be transparent
6. Assessment should stimulate positive educational effects and self-regulated learning.
8. Multiple assessment methods should be used in a comparable and standardized manner
9. Authenticity of content of assessment tasks and student work should be explicitly monitored.
10. Policies should be in place for appropriate and accurate decisions
11. Assessment processes should be cost-efficient as well as time and resources efficient
12. Policies for collection, interpretation and use of assessment data should be acceptable to all stakeholders. ( Jiang, 2011)

The performance indicators which were employed include on-time completion rates, percentage of competencies/EPAs assessed, student satisfaction rates and faculty perception indicators. An effective assessment system is expected, in the long run, to enhance student attainment of competencies, reiterating the overarching purpose of assessment to enhance student learning. Evaluation of the quality of assessment aligns with the institutional QA cycle and is part of program evaluation.

*Enabling factors for the development of the assessment plan*

Our findings on the focus of competency-based assessment align with the enabling factors reported in a recent study on assessment of surgical residents. They recommend that competency-based assessment tools should ensure comprehensive holistic evaluations of students focusing on competencies which were neglected previously. They also noted that evidence-based assessment designs and tools of high validity and reliability should be used. Their recommendation for future is that these tools should be scaled up to be applicable more widely and to further develop existing tools through research to create novel tools to enhance accuracy in future ( Hanrahan *et al.*, 2021).

We can conclude that the enabling factors which are required for success of a competency-based assessment system are:

1. Leadership committed to excellence in competency attainment
2. Clear strategy and vision to support competency-based education and assessment
3. Financial support and funding for technology, infrastructure and human resources (IT, administrative, technical, assessment experts, etc.
4. Well-defined policies with job descriptions with clear roles, responsibility and accountability to deal with a larger number of assessments and large amount of data.
5. Decision-making authority of the Academic affairs and Assessment committee. Well defined organization with clear contingency planning
6. Dedicated and committed faculty members, instructors, preceptors and assessors who are committed to CBME
7. Faculty and staff development, regularly updating and up-skilling members
8. Consultancy and partnership with best-in-class institutions for specific purposes in CBME
9. Robust CB assessment processes which are regularly updated and disseminated.
10. Clear and simple Assessment documentation structure with checks and balances.
11. Regular cycles of designing, Implementation, review and improvement. Comprehensive QA processes and effective trouble-shooting and continuous improvement.
12. Well defined grievances, appeal, disciplinary policies.
13. Proper insight into the individuality of the setting in which the plan has to be executed.

*Limitations perceived while developing the assessment plan*

The limitations in developing a competency-based assessment plan include the multiplicity of information sources and consultancy opportunities, the difficulty of aligning information and making decisions, as well as unrealistic expectations from consultants. Government mandates set time limits on the launch of the new program that results in working under pressure of time along with multitasking and having a high workload by the members of the core team. It appears to be especially challenging to run an old curriculum at the time of designing and planning a new program. The unique nature of the students being females with Islamic culture necessitates adaptations to all the resources, information, utilities and plans. We also faced a lack of detailed guidelines from accreditation bodies and lack of frame of reference. The organization itself lacks IT experts in educational technology to take the lead. Finally, we find that the transition to competency-based assessment along with the initiation of a new curriculum is a massive transformation and difficult to implement.

## Ethics

The approval # AY22-23-F-36 was issued by the Research and Ethics Committee of DMCG.

## Data Availability

No data are associated with this article.
