# Supplementary material for: Operationalizing competency-based assessment: Contextualizing for cultural and gender divides
Source: MedEdPublish (2016). 2023 Oct 11;13:210. [Version 1] doi: 10.12688/mep.19728.1 (PMC10576182; doi:10.12688/mep.19728.1)
Supplement: Peer review history

# Reviewer report: Reviewer response for version 1

**DOI:** 10.21956/mep.21136.r35013

**Published:** 2023-11-10

**Authors:** Janet grant

**Affiliations:** 1 The Centre for Medical Education in Context, Hampton, UK

This paper is an excellent, detailed, illustrated  and unusual account of how to design and manage and assessment system for a competency based curriculum. It would be of considerable interest and help to anyone having to undertake this task: as many in medical education currently are, as competency based curricula sweep medical schools acres the globe. From that point of view, this will be a key text on many Master's of Health Professions Education courses. 

 I have no recommendations for alteration of the text in terms of managing assessment. However, I would add two points for consideration:

1. The paper's title leads us to believe that the argument will address gender and culture. Although these are mentioned along the way, this aspect is not presented with evidence or argument and the conclusions do not tell us how the DCMG system has been modified to address gender and culture. I think that gender and culture are too complex to address in a paper that sets out the details of assessment management, as this one does. I would strongly recommend that this paper simply addresses the design and management of an assessment system for CBME - which is beautifully set out. And that reference to gender and culture are omitted. I would further suggest that a separate paper that does address gender and culture in relation to assessment should be invited. 
2. The paper does not address many issues in assessment such as item writing, station design, validity and reliability of scores, and standard setting. That is fine, and does not detract from the value of the paper, but this should be made clear at the outset. 

If evidence from practice is presented, are all the underlying source data available to ensure full reproducibility?

Yes

Is the topic of the practical tips discussed accurately in the context of the current literature

Yes

Are all factual statements correct and adequately supported by citations?

Partly

Are the conclusions drawn balanced and justified on the basis of the presented arguments?

Partly

Are arguments sufficiently supported by evidence from the published literature and/or the authors’ practice?

Partly

Reviewer Expertise:

Curriculum, accreditation,

I confirm that I have read this submission and believe that I have an appropriate level of expertise to confirm that it is of an acceptable scientific standard, however I have significant reservations, as outlined above.

# Reviewer report: Reviewer response for version 1

**DOI:** 10.21956/mep.21136.r35016

**Published:** 2023-11-10

**Authors:** Mohamed Elhassan Abdalla

**Affiliations:** 1 Faculty of Education & Health Services, University of Limerick, Limerick, County Limerick, Ireland

General Comment

- The article addresses an important area of how to design assessments based on the competencies agreed on.

Specific Comments

 Abstract

- The abstract introduces readers to the context and the rationale of the article. The use of the word (operationalised) may need to be explained as it may mean differently to different people.

Background

- The background could describe the national context (UAE) of the manuscript; the literature review on assessment has developed a rationale for the work presented. The paragraph on DMCG could come earlier to describe the school and should be tailored to serve the manuscript's aim; a few words on the curriculum will be of more benefit to readers. 

Content of the Manuscript

- The authors provide a detailed description of how they plan and implement assessment at DMCG. The guide can work as a thorough guide for others with similar contexts or after considering one's context.
- The tables provided are helpful; they may need more description that the codes included are the codes of the modules used in the author's medical school.
- A link to the UAE competencies will help readers to understand the EmiratesMED competency areas in table 1.
- Table 5 needs a title
- It will be of value if the authors can describe the process of getting the percentages in table 6
- 11 tables may be seen as too much but in the context of this guide, it is needed.
- A paragraph or more on the impact of implementing this process may be of value to readers and encourage others to use the guide (it may be a plan for another manuscript)
- Conclusion and take-home message is needed

If evidence from practice is presented, are all the underlying source data available to ensure full reproducibility?

Partly

Is the topic of the practical tips discussed accurately in the context of the current literature

Yes

Are all factual statements correct and adequately supported by citations?

Yes

Are the conclusions drawn balanced and justified on the basis of the presented arguments?

Yes

Are arguments sufficiently supported by evidence from the published literature and/or the authors’ practice?

Yes

Reviewer Expertise:

Medical Education, Social Accountability

I confirm that I have read this submission and believe that I have an appropriate level of expertise to confirm that it is of an acceptable scientific standard, however I have significant reservations, as outlined above.

# Reviewer report: Reviewer response for version 1

**DOI:** 10.21956/mep.21136.r35014

**Published:** 2023-10-16

**Authors:** Ciraj Ali Mohammed

**Affiliations:** 1 Microbiology Department, Manipal Academy of Higher Education, Manipal, Karnataka, India

This research focuses on operational aspects of contextualizing competency assessment and elucidates the ways adopted in building the team, working with consultants and harnessing faculty development in this area. The maps generated are an outcome of this study and linked to institutional assessment capacity, and a blended effort using both human capital and technology. I feel they must give more clarity on mapped competency providing evidence that the physicians being trained are fit for practice. 

 The enabling factors that helped in the execution is well detailed. The work also points out the challenges they faced in the process of implementation. However arguments are not adequately supported by evidence from the published literature. 

 Operationalizing competency-based assessment is an ongoing process and hence requires a commitment to continuous improvement and a focus on authentic assessments that support student learning. I hope the authors would add a few words on these initiatives.

If evidence from practice is presented, are all the underlying source data available to ensure full reproducibility?

Partly

Is the topic of the practical tips discussed accurately in the context of the current literature

Yes

Are all factual statements correct and adequately supported by citations?

Yes

Are the conclusions drawn balanced and justified on the basis of the presented arguments?

Yes

Are arguments sufficiently supported by evidence from the published literature and/or the authors’ practice?

Partly

Reviewer Expertise:

Interprofessional education, Competency based medical education, Assessment, Academic leadership, Problem based learning

I confirm that I have read this submission and believe that I have an appropriate level of expertise to confirm that it is of an acceptable scientific standard, however I have significant reservations, as outlined above.

# Reviewer report: Reviewer response for version 1

**DOI:** 10.21956/mep.21136.r35017

**Published:** 2023-10-13

**Authors:** Nagwa Nashaat

**Affiliations:** 1 Family Medicine Department, Menoufia University, Shebeen El-Kom, Menofia Governorate, Egypt

This is indeed a timely, relevant, and important topic and will be of interest to the researchers in the qualitative field. The manuscript is easy to read. There is a clear statement of the problem and purpose of the study.

 The authors have done a commendable job of developing a conceptual framework that guides the implementation of competency-based assessment in medical education. They have outlined the following steps in their framework:

- Preparing the institution for the change by assessing its capacity, developing the faculty, and benchmarking with other institutions.
- Mapping the course learning outcomes (CLOs) to the program learning outcomes (PLOs), and mapping the competencies and the PLOs to the courses, ensuring alignment with the UAE-specific requirements.
- Executing the assessment plan by revising the existing assessment tools and introducing new ones that match the competencies, and determining the assessment principles and weightings for each competency across the curriculum.
- Using software to facilitate secure and efficient computer-based assessment, and providing training for faculty on how to use the system.
- Evaluating the quality and validity of the assessment tools and their impact on student learning and performance.

In short:  The title includes the key concepts the research is based on. The abstract puts the research into context (general and specific background). The introduction is clear and well-organized. A clear conceptual framework that guides the implementation of competency-based assessment in medical education. References are relevant.

 This work is a valuable resource for medical educators who want to adopt a competency-based approach to assessment in their programs.

If evidence from practice is presented, are all the underlying source data available to ensure full reproducibility?

Not applicable

Is the topic of the practical tips discussed accurately in the context of the current literature

Yes

Are all factual statements correct and adequately supported by citations?

Yes

Are the conclusions drawn balanced and justified on the basis of the presented arguments?

Yes

Are arguments sufficiently supported by evidence from the published literature and/or the authors’ practice?

Yes

Reviewer Expertise:

Curriculum design, Program evaluation, Assessment

I confirm that I have read this submission and believe that I have an appropriate level of expertise to confirm that it is of an acceptable scientific standard.

# Reviewer report: Reviewer response for version 1

**DOI:** 10.21956/mep.21136.r35012

**Published:** 2023-10-12

**Authors:** Dalia Ali

**Affiliations:** 1 Associate Professor of Medical Biochemistry & Molecular Biology, Gulf Medical University, Ajman, United Arab Emirates

I would like to thank the authors for undertaking this mission of developing this conceptual framework which provides a complete step-by-step guide to competency-based assessment.

 The preparation phase clearly describes how the institution's capacity was first assessed. In my opinion, the most important steps were: faculty development and the benchmarking process.

 In the mapping section, the authors mentioned clearly the complete phases of mapping; CLOs to PLOs, competencies to the courses, and PLOs to the courses. This is of particular importance in the sense that the taught courses & topics within are all relevant and aligned to the UAE requirements for competencies in medical schools.

 Then, comes the execution part, where all steps should be revised before being put into action. The assessment plan is revised with the introduction of new assessment tools that abide by the newly introduced competencies.

 The authors also determined the assessment principles for each competency and how much each assessment tool contributes to the total competency assessment taking into consideration that a higher level of competency assessment will be attained in the clinical phase.

 The role of the software in accomplishing a secure and efficient computer-based assessment is emphasized. In this phase, faculty development workshops on the use of the SIS platforms are necessary.

 Finally, the evaluation phase is very important to measure the quality of all the assessment tools.

 In my opinion, this work provides a remarkable guide for medical educators on the detailed plan that can be adopted to fulfill competency- based assessment in medical schools.

If evidence from practice is presented, are all the underlying source data available to ensure full reproducibility?

Not applicable

Is the topic of the practical tips discussed accurately in the context of the current literature

Yes

Are all factual statements correct and adequately supported by citations?

Yes

Are the conclusions drawn balanced and justified on the basis of the presented arguments?

Yes

Are arguments sufficiently supported by evidence from the published literature and/or the authors’ practice?

Yes

Reviewer Expertise:

Assessment, Student engagement, curriculum development.

I confirm that I have read this submission and believe that I have an appropriate level of expertise to confirm that it is of an acceptable scientific standard.
